# Supplementary material for: Perception, knowledge, and attitude of medical doctors in Saudi Arabia about the role of physiotherapists in vestibular rehabilitation: a cross-sectional survey
Source: PeerJ. 2022 Mar 7;10:e13035. doi: 10.7717/peerj.13035 (PMC8908887; doi:10.7717/peerj.13035)
Supplement: Appendix S2 [file peerj-10-13035-s002.pdf]

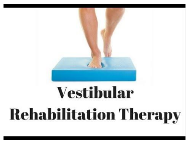

Introduction

You are welcome to participate in our study about your perception of the role of the physiotherapist in vestibular rehabilitation.

You are eligible to participate if you are a medical doctor currently practicing in the kingdom of Saudi Arabia.

There are just 23 questions, which will take only 10 minutes of your time.

Confidentiality:

Your answers will be protected and will maintain the privacy of your information. The researchers will not disclose or share your information with anyone.

Your participation is highly appreciated

Researcher,

Dr. Danah Alyahya

Assistant Professor at Majmaah University

Email: d.alyahya@mu.edu.sa

Mr. Faizan Zaffar Kashoo

Lecture at Majmaah University

Email: f.kashoo@mu.edu.sa

This research had Ethical approval from Majmaah University

Ethics Number: MUREC -April.7/COM-2021/31-1

The study aims to evaluate the knowledge, amount of referrals, perception, and attitude of physicians towards the role of physical therapists in vestibular rehabilitation.

1- Are you willing to participate in the survey?

- Yes
- No

2- Your age?

- ○ 24 – 30 years old
- ○ 31 – 40 years old
- ○ 41 – 50 years old
- ○ 51 – 60 years old
- ○ Above 60

3- Your gender?

- ○ Male
- ○ Female

4- Nationality

[ ]

5- What is your Specialty as a medical doctor?

- ○ General Physicians
- ○ Pediatricians
- ○ General Surgeon
- ○ Cardiologist
- ○ Dentist
- ○ Dermatologists
- ○ Gynecologist
- ○ ENT Specialist
- ○ Neurology
- ○ Family physician
- ○ Psychiatrist
- ○ [ ] Others, please specify

6- Last professional degree obtained from

[ ]

7- Years of experience do you have in medical field?

- 1 – 3 years
- 4 – 8 years
- 9 – 15 years
- More than 15 years

8- Which of the following province you are currently practicing in?

- Riyadh
- Makkah
- Eastern
- Madinah
- Al Baha
- Al Jawf
- Northern Borders
- Qassim
- Ha'il
- Tabuk
- Aseer
- Jazan
- Najran

9- **Working Place?**

- Governmental Hospital
- Private hospital
- Clinic
- ______________ Others

10- **Did you see patients with Vestibular Disorder?**

- Yes
- No

11- How many patients with vestibular disorder did you see per month?

- ○ Less than 5 patients
- ○ 5 – 10 patients
- ○ 11 – 20 patients
- ○ More than 20 patients

12- What is the most common cases that you diagnosed with vestibular disorder? (You can choose more than one).

- ☐ Benign Paroxysmal Positional Vertigo (BPPV)
- ☐ Vestibular Neuritis
- ☐ Unilateral/Bilateral Vestibular Hypofunction
- ☐ Vestibular Migraine
- ☐ Meniere's Disease
- ☐ Vestibular Paroxysmal
- ☐ Mal de Debarquement
- ☐ Perilymphatic Fistula
- ☐ Cervicogenic Dizziness
- ☐ Traumatic Brain Injury
- ☐ Motion Sensitivity
- ☐ Multiple Sclerosis
- ☐ ________ Others

13- Who do you think can provide better vestibular rehabilitation? (You can choose more than one option)

- ☐ My self
- ☐ ENT
- ☐ Audiologist
- ☐ Physical therapist
- ☐ Occupational therapist
- ☐ Nurses
- ☐ ________ Others, please specify

14- Are you aware that physiotherapists are trained to provide vestibular rehabilitation?

- ○ Yes

- No

15- Do you think physiotherapists are able to assess and diagnose patients with vestibular disorders?

- Yes
- No

16- Why do you think physiotherapist can NOT assess and diagnose the patient with vestibular disorders? (you can choose more than one)

- Not applicable
- Not aware that physiotherapy can evaluate vestibular disorder patients
- Insufficient of knowledge
- Less confidence
- Less experience
- ________ Others

17- Do you think physiotherapists will be able to treat patients with vestibular disorders?

- Yes
- No

18- Why do you think physiotherapist can NOT treat the patient with vestibular disorders? (you can choose more than one)

- Not applicable
- Not aware that physiotherapy can evaluate vestibular disorder patients
- Insufficient of knowledge
- Less confidence
- Less experience
- ________ Others

19- What type of patients do you refer to physiotherapist? (You can choose more than one).

- ☐ None.
- ☐ Benign Paroxysmal Positional Vertigo (BPPV)
- ☐ Vestibular Neuritis
- ☐ Unilateral/Bilateral Vestibular Hypofunction
- ☐ Vestibular Migraine
- ☐ Meniere’s Disease
- ☐ Vestibular Paroxysmal
- ☐ Mal de Debarquement
- ☐ Perilymphatic Fistula
- ☐ Cervicogenic Dizziness
- ☐ Traumatic Brain Injury
- ☐ Motion Sensitivity
- ☐ Multiple Sclerosis
- ☐ ________ Others

20. Rate the physiotherapist involvement in the management of the following disorders

	No role	Involvement in assessment	Involvement in assessment and treatment	Haven't seen the cases
Benign Paroxysmal Positional Vertigo (BPPV)	○	○	○	○
Vestibular Neuritis	○	○	○	○
Unilateral/Bilateral Vestibular Hypofunction	○	○	○	○
Vestibular Migraine	○	○	○	○
Meniere’s Disease	○	○	○	○
Vestibular Paroxysmal	○	○	○	○
Mal de Debarquement	○	○	○	○
Perilymphatic Fistula	○	○	○	○
Cervicogenic Dizziness	○	○	○	○
Traumatic Brain Injury	○	○	○	○
Motion Sensitivity	○	○	○	○
Multiple Sclerosis	○	○	○	○

21- Which of the following assessment method do you think physiotherapists can apply effectively? (You can choose more than one).

- ☐ None
- ☐ Dix-Hallpick test
- ☐ Roll test
- ☐ Side-lying test
- ☐ Head Impulse Test/ Head Thrust Test
- ☐ Head Shaking Nystagmus Test
- ☐ Dynamic Visual Acuity Test
- ☐ Oculoomotor examination
- ☐ VOR Cancellation
- ☐ Modified Clinical Test of Sensory Interaction and Balance
- ☐ I am not aware about these tests
- ☐ ________ Others

22- Which of the following treatment method do you think physiotherapists can apply effectively? (You can choose more than one)

- ☐ None
- ☐ Canalith Repositioning Maneuver / (Ebly) Maneuver
- ☐ Semont Maneuver
- ☐ Gufoni / Casani Maneuver
- ☐ Barbecue Roll Maneuver
- ☐ Imaginary Target Exercise
- ☐ Vestibulo-ocular reflex Adaptation 1 (VOR 1)
- ☐ Vestibulo-ocular reflex Adaptation 2 (VOR 2)
- ☐ Substitution Exercises: Active Eye Movements Between Two Targets
- ☐ Habituation Exercise (Brandt - Daroff Maneuver)
- ☐ I am not aware about these treatment methods.
- ☐ I am not sure about the effectiveness of these treatment methods.
- ☐ ________ Others

23- Did you ever get feedback from the patients after your referral to a physiotherapist? What was the patient feedback?

- ☐ Never got feedback from patients
- ☐ Not satisfied
- ☐ Moderately satisfied
- ☐ Highly satisfied

Powered by Qualtrics
