# Supplementary material for: Perception, knowledge, and attitude of medical doctors in Saudi Arabia about the role of physiotherapists in vestibular rehabilitation: a cross-sectional survey
Source: PeerJ. 2022 Mar 7;10:e13035. doi: 10.7717/peerj.13035 (PMC8908887; doi:10.7717/peerj.13035)
Supplement: Supplemental Information 3 [file peerj-10-13035-s003.docx]

Number of mutually inclusive responses (n=177) from the medical doctors related to reason why physiotherapist does not have role in assessment and diagnosis of patients with vestibular rehabilitation.

| **Response from Medical Doctor** | **Item 1** | **Item 2** | **Item 3** | **Item 4** | **Sum of responses** |
| --- | --- | --- | --- | --- | --- |
| **Item 1** | 156 | 4 | 6 | 0 | 156 |
| **Item 2** | 0 | 2 | 2 | 5 | 5 |
| **Item 3** | 6 | 6 | 10 | 2 | 10 |
| **Item 4** | 4 | 6 | 6 | 2 | 6 |
|  | 156 | 6 | 10 | 5 | **177** |

**Item 1:** Not Aware about the role of Physiotherapist in the assessment and diagnosis of patient with vestibular rehabilitation.

**Item 2:** Less experience in the assessment and diagnosis of patient with vestibular rehabilitation.

**Item 3:** Less confidence in the assessment and diagnosis of patient with vestibular rehabilitation.

**Item 4:** Insufficient knowledge in assessment and diagnosis patients with vestibular rehabilitation.

Number of mutually inclusive responses (n=156) from the medical doctors related to reason why physiotherapist does not have role in treatment of patients with vestibular rehabilitation.

| **Response from Medical Doctor** | **Item 1** | **Item 2** | **Item 3** | **Sum of responses** |
| --- | --- | --- | --- | --- |
| **Item 1** | 145 | 0 | 0 | 145 |
| **Item 2** | 0 | 9 | 1 | 9 |
| **Item 3** | 0 | 1 | 2 | 2 |
|  | 145 | 9 | 2 | **156** |

**Item 1:** Not Aware about the role of Physiotherapist in the treatment of patient with vestibular rehabilitation.

**Item 2:** Less experience in the treatment of patient with vestibular rehabilitation.

**Item 3:** Insufficient knowledge to treat patients with vestibular rehabilitation.
